# Supplementary material for: Establishment of Neurospora crassa as a model organism for fungal virology
Source: Nat Commun. 2020 Nov 6;11:5627. doi: 10.1038/s41467-020-19355-y (PMC7648066; doi:10.1038/s41467-020-19355-y)
Supplement: Supplementary file 1 — Supplementary Information [file 41467_2020_19355_MOESM1_ESM.pdf]

Supplementary Information

Establishment of *Neurospora crassa* as a model organism for fungal virology

Honda et al.

Supplementary Figures 5

Supplementary Tables 4

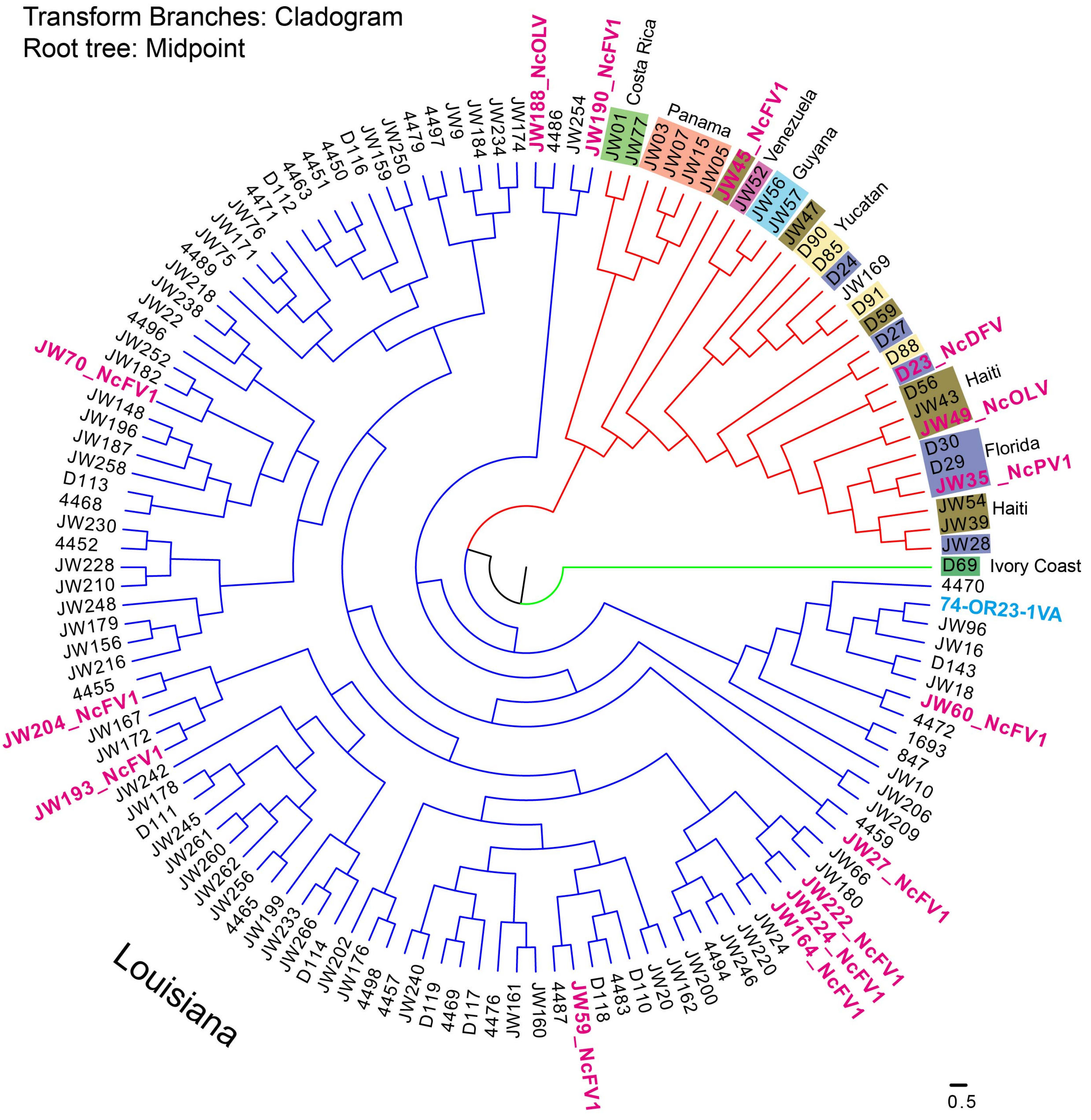

Supplementary Figure 1. Cladogram of *Neurospora crassa* strains and detected viruses. The cladogram was constructed based on single nucleotide polymorphisms (SNPs) identified from the available transcriptomic data in wild *N. crassa* strains using Snippy and RAxML-NG. A GTR+G4 model was selected as a best-fit model for the alignment using ModelTest-NG. The trees are visualized by FigTree with the midpoint rooting method. The stock name, geographic origin and infected virus name (see Table 1 and Supplementary Table 1) in wild *N. crassa* strains are shown.

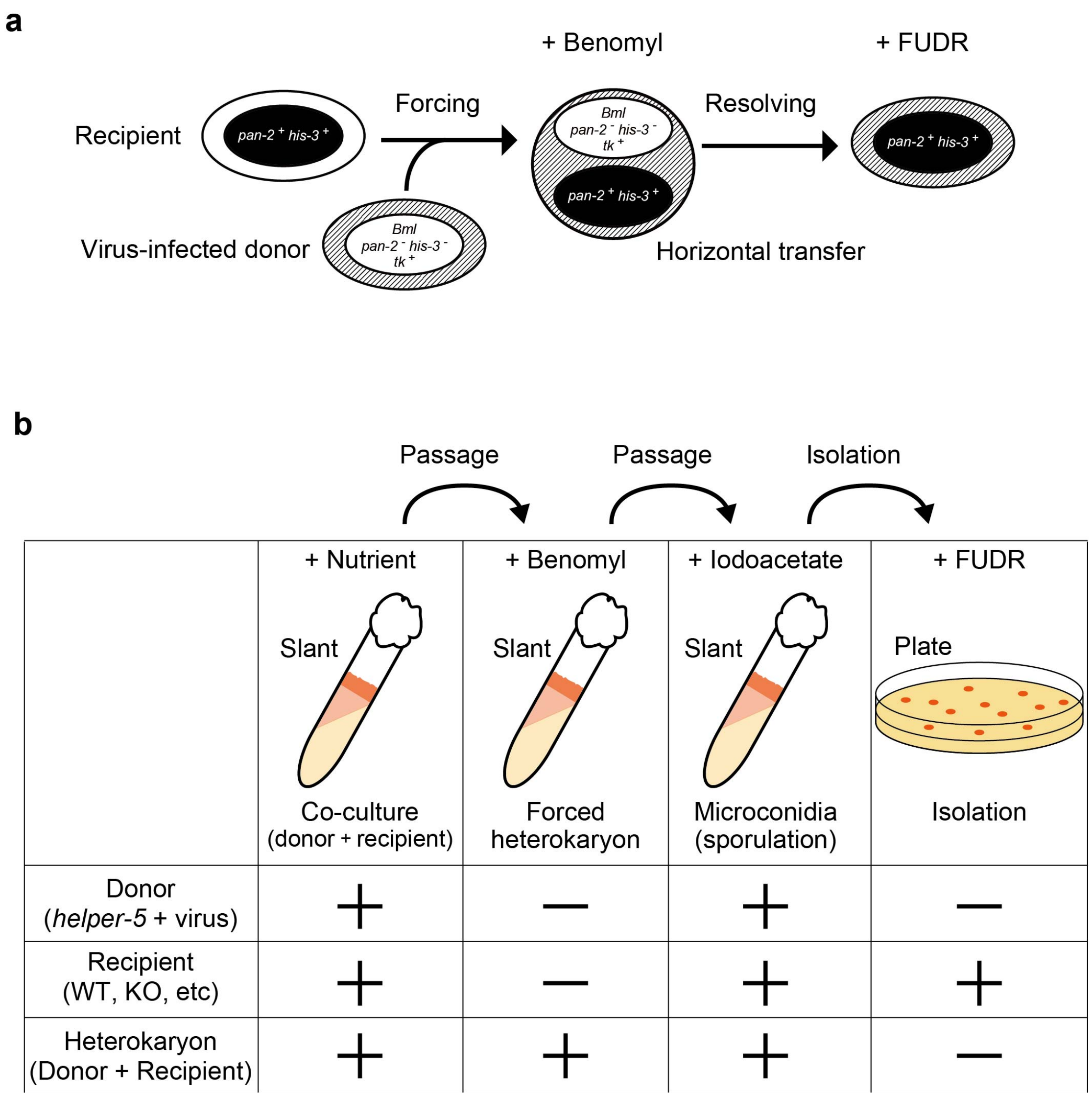

	+ Nutrient (Co-culture (donor + recipient))	+ Benomyl (Forced heterokaryon)	+ Iodoacetate (Microconidia (sporulation))	+ FUDR (Isolation)
Donor (*helper-5* + virus)	+	−	+	−
Recipient (WT, KO, etc)	+	−	+	+
Heterokaryon (Donor + Recipient)	+	+	+	−

Supplementary Figure 2. The *helper-5* strain-mediated horizontal transfer of viruses. (a) Schematic representation showing essential genes and medium conditions for virus transmission. A recipient strain (open circle) forces heterokaryon formation with the virus-infected *helper-5* strain (cross-hatched circle) on medium containing Benomyl but lacking histidine and pantothenate. Only heterokaryotic cells can survive these culture media, which have the nutritional markers *his-3*$^{+}$ and *pan-2*$^{+}$ and the dominant Benomyl resistance gene *Bml*. The resulting virus-infected recipient strain was resolved using the *helper-5 tk*$^{+}$ which is the herpes simplex virus thymidine kinase gene, conferring 5-fluorouracil-2′-deoxyriboside (FUDR) sensitivity to *N. crassa*. In addition, the *helper-5* strain lacking *mat* has lost heterokaryon incompatibility specified by mating type, thus being able to form heterokaryon with any recipient strains with either mating type *A* or *a*. **(b)** Schematic diagram showing four steps, co-culture, forced heterokaryon, microconidiation and isolation, for virus introduction into recipient strains. The table shows that donor, recipient and heterokaryon cells were able (+) or unable (-) to grow onto medium containing nutrient, Benomyl, iodoacetate or FUDR.

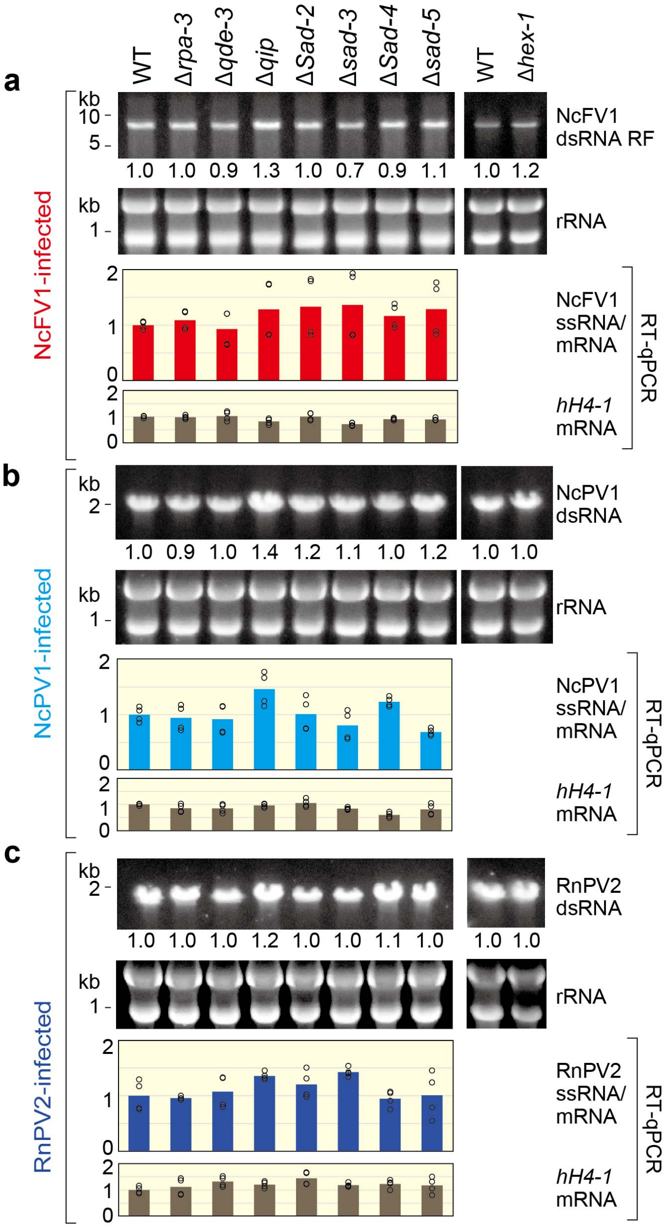

Supplementary Figure 3. Quantitative comparison of virus accumulation in different mutants of *Neurospora crassa*. Accumulation of Neurospora crassa fusarivirus 1 (NcFV1) **(a)**, Neurospora crassa partitivirus 1 (NcPV1) **(b)**, and Rosellinia necatrix partitivirus 2 (RnPV2) **(c)** in *N. crassa* single deletion mutants of RNA silencing-related genes. The mutants have the same genetic background as the *N. crassa* standard strain, 74-OR23-1VA. After normalized against ribosomal RNA (rRNA) as shown on the middle panels, the obtained dsRNA fractions were electrophoresed. Virus accumulation was quantified by comparing band intensity of the viral dsRNA replicative form (RF) bands and normalizing against rRNA using ImageJ. Mean values obtained from two biological replicates are shown below each gel, where virus accumulation in the wild type strain was expressed as 1. Accumulation of positive-sense (+) single-stranded (ss) RNA of the three viruses was also compared among the aforementioned *N. crassa* mutants by real-time qPCR. The histograms below the gels show averaged relative accumulation on the measurements of two biological replicates and two technical replicates. A total of four obtained values are shown by open circles on each bar.

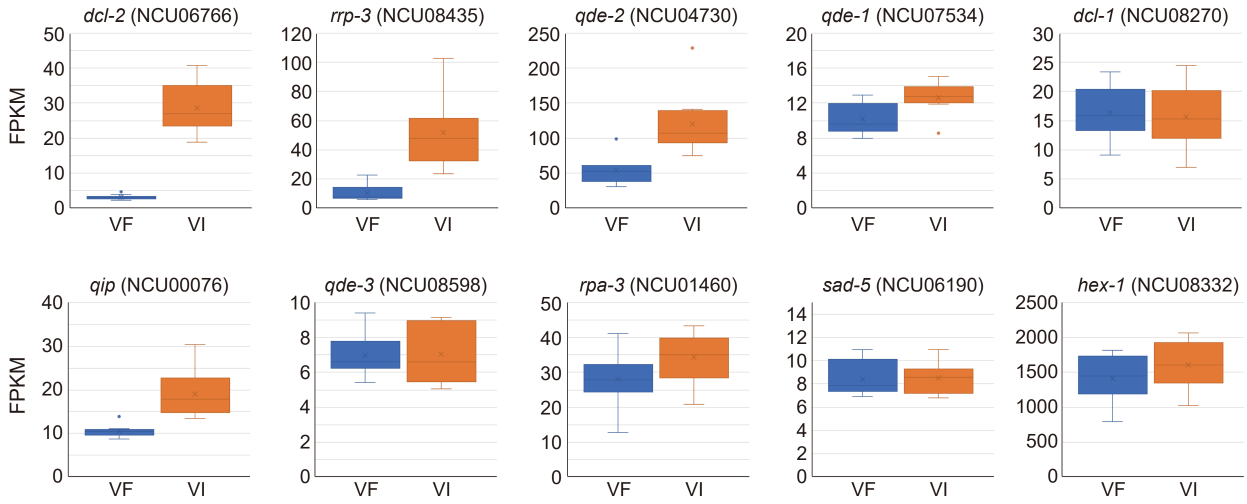

Supplementary Figure 4. Differential gene expression (DEG) analysis of RNAi related genes in *N. crassa*. Transcriptomic data of ten each of the virus-infected (VI) and highly likely virus-free (VF) *N. crassa* strains (see Methods and Supplementary Table 1) were compared. Ten genes shown on the top of each graph were compared among 10 virus-infected and 10 virus-free transcriptomic data sets (see Methods). Their transcript levels, expressed by FPKM (fragments per kilobase per million reads mapped), are shown by conventional boxplots that illustrate five-number summaries including medians denoted by horizontal lines and interquartile range indicated by boxes.

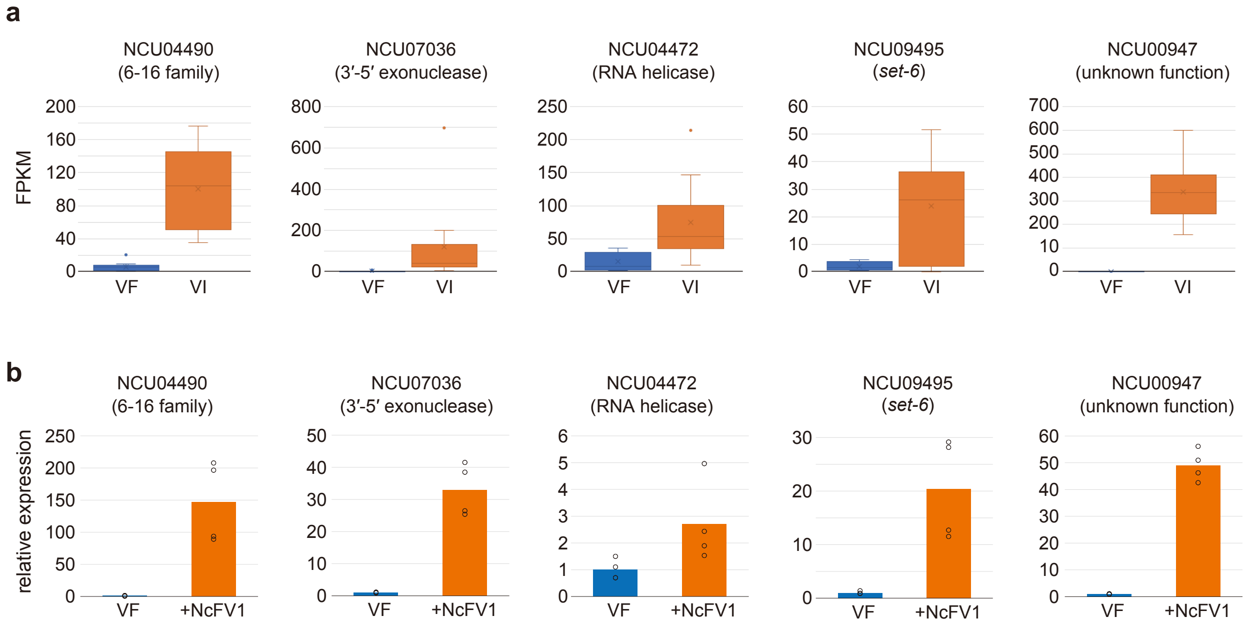

Supplementary Figure 5. Transcriptional up-regulation of dsRNA-inducible genes in *N. crassa*. Transcript levels of five representative dsRNA-inducible genes shown on the top of each graph were compared between virus-infected (VI) and highly likely virus-free (VF) *N. crassa* strains taking two approaches. **(a)** In differential gene expression (DEG) analysis, transcript levels, shown by FPKM, were compared by boxplots as in Supplementary Fig. 4. The same transcriptomic data sets previously reported by other research groups were utilized (see Supplementary Table 1). **(b)** Transcript levels of the five genes indicated on the top of each graph were compared by RT-qPCR between a virus-free (VF) standard *N. crassa* strain (74-OR23-1VA) and its NcFV1-infected strain. Mean values shown by bars were determined from two biological replicates and two technical replicates for each. Obtained values are indicated by open circles (four circles placed on each bar). Histone H4 mRNA (*hH4-1*) was used as an internal control.

Supplementary Table 1. List of BioProjects and SRA datasets analyzed in this study

BioProject	SRA file	strain name	*in silico* detection*
N.crassa			
	SRR080687	D69	
	SRR089764	D23	
	SRR089765	D24	
	SRR089766	D27	
	SRR089767	D29	
	SRR089768	D30	
	SRR089769	D56	
	SRR089771	D59	
	SRR089778	D85	
	SRR089779	D88	
	SRR089780	D90	
	SRR089781	D91	
	SRR089782	JW01	
	SRR089783	JW03	
SRP004848	SRR089825	JW05	
	SRR089826	JW07	
	SRR089831	JW15	
	SRR089834	JW28	
	SRR089835	JW35	virus-detected
	SRR089836	JW39	
	SRR089837	JW43	
	SRR089840	JW45	virus-detected
	SRR089842	JW47	
	SRR089843	JW49	virus-detected
	SRR089844	JW52	
	SRR089845	JW54	
	SRR089846	JW56	
	SRR090355	JW57	
	SRR090366	JW77	
	SRR797950	FGSC #2489	potentally virus-free
	SRR797951	FGSC #1693	potentally virus-free
	SRR797954	FGSC #4450	potentally virus-free
	SRR797955	FGSC #4451	potentally virus-free
	SRR797956	FGSC #4452	potentally virus-free
	SRR797958	FGSC #4455	
	SRR797959	FGSC #4457	
	SRR797960	FGSC #4459	
	SRR797961	FGSC #4463	potentally virus-free
	SRR797962	FGSC #4465	potentally virus-free
	SRR797963	FGSC #4468	
	SRR797964	FGSC #4469	potentally virus-free
	SRR797965	FGSC #4470	potentally virus-free
	SRR797966	FGSC #4471	
	SRR797967	FGSC #4472	potentally virus-free
	SRR797968	FGSC #4476	
	SRR797969	FGSC #4479	
	SRR797970	FGSC #4483	
	SRR797971	FGSC #4486	
	SRR797972	FGSC #4487	
	SRR797973	FGSC #4489	
	SRR797974	FGSC #4494	
	SRR797975	FGSC #4496	
	SRR797976	FGSC #4497	
	SRR797977	FGSC #4498	
	SRR797979	FGSC #847	
	SRR797980	D110	
	SRR797981	D111	
	SRR797982	D112	
	SRR797983	D113	
	SRR797984	D114	
	SRR797985	D116	
	SRR797986	D117	
	SRR797987	D118	
	SRR797988	D119	
	SRR797989	D143	
	SRR797990	JW10	
	SRR797991	JW148	
	SRR797992	JW156	
	SRR797993	JW159	
	SRR797994	JW16	
	SRR797995	JW160	

PRJNA194554	SRR797996	JW161	
	SRR797997	JW162	
	SRR797998	JW164	virus-detected
	SRR797999	JW167	
	SRR798001	JW169	
	SRR798002	JW171	
	SRR798003	JW172	
	SRR798004	JW174	
	SRR798005	JW176	
	SRR798006	JW178	
	SRR798007	JW179	
	SRR798008	JW18	
	SRR798009	JW180	
	SRR798010	JW182	
	SRR798011	JW184	
	SRR798012	JW187	
	SRR798013	JW188	virus-detected
	SRR798014	JW190	virus-detected
	SRR798015	JW193	virus-detected
	SRR798016	JW196	
	SRR798017	JW199	
	SRR798018	JW20	
	SRR798019	JW200	
	SRR798020	JW202	
	SRR798021	JW204	virus-detected
	SRR798022	JW206	
	SRR798023	JW209	
	SRR798024	JW210	
	SRR798025	JW216	
	SRR798026	JW218	
	SRR798027	JW22	
	SRR798028	JW220	
	SRR798029	JW222	virus-detected
	SRR798030	JW224	virus-detected
	SRR798031	JW228	
	SRR798032	JW230	
	SRR798033	JW233	
	SRR798034	JW234	
	SRR798035	JW238	
	SRR798036	JW24	
	SRR798037	JW240	
	SRR798038	JW242	
	SRR798039	JW245	
	SRR798040	JW246	
	SRR798041	JW248	
	SRR798042	JW250	
	SRR798043	JW252	
	SRR798044	JW254	
	SRR798045	JW256	
	SRR798046	JW258	
	SRR798047	JW260	
	SRR798048	JW261	
	SRR798049	JW262	
	SRR798050	JW266	
	SRR798051	JW27	virus-detected
	SRR798053	JW59	virus-detected
	SRR798054	JW60	virus-detected
	SRR798055	JW66	
	SRR798057	JW70	virus-detected
	SRR798058	JW75	
	SRR798059	JW76	
	SRR798060	JW9	
	SRR798061	JW96	
N. discreta			
PRJNA257829	SRR1539773	FGSC #8579	virus-detected
	SRR1539774	FGSC #8578 x FGSC #8579	
	SRR1539775	FGSC #8578 x FGSC #8579	
	SRR1539776	FGSC #8578 x FGSC #8579	
	SRR1539777	FGSC #8578 x FGSC #8579	
	SRR1539778	FGSC #8578 x FGSC #8579	
	SRR1539779	FGSC #8578 x FGSC #8579	
	SRR1539780	FGSC #8578 x FGSC #8579	

*: 10 virus-infected strains (highlighted light blue) and 10 highly likely virus-free strains (highlighted faint red) were sellected for differential gene expression (DEG) analysis in Supplementary Figs. 4 and 5a. See also Table 1.

Supplementary Table 2. List of *Neurospora crassa* strians

N. crassa standard strain (74-OR23-1VA strain)	FGSC strain name	virus-free	+NcFV1	+NcPV1	+RnPV2
**Wild type**					
mat A	FGSC #2489	N3752	#4779	#4846	#4780
RDR mutants					
mat a, Δqde-1::hph	FGSC #11156	#4494	#4504	#4812	#5584
mat a ΔSad-1::hph	FGSC #11151	#357	#5150	#5151	#5585
mat a, Δrrp-3::hph	FGSC #11670	#4945	#4922	#4925	#5586
mat A ΔSad-1::hph, Δqde-1::nat, Δrrp-3::hph	this study	#5096	#5099	#5152	#5587
Dicer mutants					
mat A, Δdcl-1::hph	FGSC #15891	#4311	#4500	#4802	#5588
mat a, Δdcl-2::hph	FGSC #11155	#1386	#4513	#4799	#5589
mat a, Δdcl-1::hph, Δdcl-2::nat	this study	#4872	#4880	#4885	#5590
Argonaute mutants					
mat A, Δqde-2::nat	this study	#4697	#4742	#4853	#5591
mat A, Δsms-2::hph	FGSC #11161	#4314	#4501	#4804	#5592
mat A, Δsms-2::hph, Δqde-2::nat	this study	#4950	#5123	#5154	#5593
other RNAi mutants					
mat A, Δrpa-3::hph	FGSC #12799	#5316	#4876	#4879	#5594
mat a Δqde-3::hph	FGSC #12505 (backcross)	#2386	#4505	#4800	#5595
mat a, Δqip::hph	FGSC #12130	#4318	#4503	#4808	#5596
mat a, ΔSad-2::hph	FGSC #20680	#4319	#4509	#4809	#5597
mat a, Δsad-3::hph	FGSC #19729	#4315	#4508	#4805	#5598
mat a, ΔSad-4::hph	FGSC #13237	#4320	#4510	#4810	#5599
mat A, Δsad-5::hph	FGSC #17863	#4317	#4502	#4807	#5600
hex-1 mutants					
mat a Δhex-1::nat	this study	#4699	#4750	#4854	#5601
FLAG-tagged strains					
mat A, rrp-3-3xFLAG::hph	this study	#5042	#5078	#5165	#5602
mat A, dcl-2-3xFLAG::hph	this study	#5048	#5081	#5166	#5603
mat a, qde-2-3xFLAG::hph	this study	#5054	#5084	#5163	#5604
helper strains					
mat-, his-3, pan-2^{-}::hph^{+}::tk^{+}, Bml, cyh-1	FGSC #8747; helper-5	#8747	#4490	#4763	#4515

SupplementaryTable 3. List of primes

**RT (qPCR)**		
dcl-2 (110 bp)		
#5964	dcl-2m qPCR FP2	CTCACTGCTTCGGCATTATTTCCA
#5965	dcl-2m qPCR RP2	AGGCCAAAATCGCGAGATGC
qde2 (109 bp)		
#5966	qde-2m qPCR FP1	CCACCGGATGTCTACAAGGGA
#5967	qde-2m qPCR RP1	CCAGATTGTCGCTGACGTGC
rrp-3 (113 bp)		
#5972	rrp-3m qPCR FP2	TCGGCCTGATTTCCTGGTGA
#5973	rrp-3m qPCR RP2	GCGGTGTGTCGTCTCCATCA
qde-1 (114 bp)		
#5976	qde-1m qPCR FP2	GTAATCAGGCCCGATCTCCCA
#5977	qde-1m qPCR RP2	GGAGGCCCAGGTTGTAGTCG
dcl-1 (108 bp)		
#5984	dcl-1m qPCR FP2	TGGATACAGGATCCGGAAAGACTC
#5985	dcl-1m qPCR RP2	AGAAGGCAATCCGTCTGGGT
hH4-1 (87 bp)		
#5988	hH4-1m qPCR FP2	TCCCAAACGACCGGCTTCAG
#5989	hH4-1m qPCR RP2	CCTTGCCGCGTCCAGTCATTTT
hex-1 (96 bp)		
#6022	hex1m qPCR FP1	CTACTACGACGACGACGCTCA
#6023	hex1m qPCR RP1	GTGACAGTCTGGGAAGCAGA
NcPV1 (106 bp)		
#5999	NcPV1 qPCR FP2	ACGTGCACTCGGTTTAGCCT
#6000	NcPV1 qPCR RP2	TGAAGACACTGGGACGTCGG
NcFV1 (89 bp)		
#6001	NcFV1 qPCR FP1	GACATCGGAAGGGCGGTACA
#6002	NcFV1 qPCR RP1	CACCCCAAGTGGCATGAACG
RnPV2 (81 bp)		
#6025	RnPV2 qPCR FP1	GCGCTCAGAAAGCACCTCAC
#6026	RnPV2 qPCR RP1	AGCGTCTTCATTCCAGGGGG
**ChIP (qPCR)**		
dcl-2 (86 bp)		
#5832	dcl-2 qPCR FP2	CAGACTGATGACCGAGGCGT
#5833	dcl-2 qPCR RP2	TTAGCGGGCTTGCCGTAAGA
qde2-1 (71 bp)		
#5836	qde-2 qPCR FP1	CCGCATCGGAGGAGTAGTCG
#5837	qde-2 qPCR RP1	GCCGCTACTTTGTCGCACTC
qde2-2 (128 bp)		
#5864	qde-2 qPCR FP6	TTGAGGTGACGTGGACCGAG
#5865	qde-2 qPCR RP6	TGTCGTGAGCTCGTTCAGCA
rrp-3-1 (117 bp)		
#5844	rrp-3 qPCR FP2	AGGCCTCTCGCATAAGGCAG
#5845	rrp-3 qPCR RP2	TGTCAGTCGTTTACCGGCGT
rrp-3-2 (147 bp)		
#5846	rrp-3 qPCR FP3	GAATGCAGGGTCAGCAAGCC
#5847	rrp-3 qPCR RP3	TAAAGTTGGCGTGCGTGCAG
qde-1 (83 bp)		
#5848	qde-1 qPCR FP1	GTGCCTATTGCGAGCGCTTT
#5849	qde-1 qPCR RP1	CGTCTATGACCTGTCGCCGT
dcl-1 (88 bp)		
#5870	dcl-1 qPCR FP3	AGCACCAGGAAACTCTGGCA
#5871	dcl-1 qPCR RP3	CCAATAGCGGGCAACACGTC
hH4-1 (106 bp)		
#290	hH4-1 RT-LP2	AGACCCGTGGTGTCCTCAAG
#291	hH4-1 RT-RP2	TAGACAACGTCGAGGGAGGTG
**KO constructs**		
Δqde-1::nat		
#5530	qde-1 KO FP1	GATTCAGCAGCGAACTGACC
#5531	qde-1 KO RP2	CCCAAAAAAATGCTCCTTCAATATCAGTTGGAGTATACAACCGGGTTAGC
#5532	qde-1 KO FP3	CGCTCTACATGAGCATGCCCTGCCCCTGAGAGATATTTGGGTGCTGTGC
#5533	qde-1 KO RP4	GTTGCTTCCGATGTGATCAGG
Δqde-2::nat		
#5557	qde-2 KO FP1	CACAGCGAGACAGTAACAGG
#5558	qde-2 KO-nat RP2	CCCAAAAAAATGCTCCTTCAATATCAGTTGAAGCCGCTACTTTGTCGCAC

#5559	qde-2 KO-nat FP3	CGCTCTACATGAGCATGCCCTGCCCCTGAGGGCTTCTAGTTTCTACAGC
#5560	qde-2 KO RP4	ACAACTGGAACAGCTTTGTCG
Δ dcl-2::nat		
#5363	DCL-2 KO FP1	TGTCTCTGTGGCGAAACACG
#5528	DCL-2 KO RP2	CCCAAAAAATGCTCCTTCAATATCAGTTGAGCCCTCTATGGTAGATACG
#5529	DCL-2 KO FP3	CGCTCTACATGAGCATGCCCTGCCCCTGACAAGGGACAGTCTCTACTCC
#5366	DCL-2 KO RP4	AGTCCTTGTAAGGATGTGTGG
Δ hex-1::nat		
#5564	hex-1 KO FP1	TATCCATACCCACAACCATGG
#5565	hex-1 KO-nat RP2	CCCAAAAAATGCTCCTTCAATATCAGTTGCAGTAGACTCGCTGTAGAGG
#5566	hex-1 KO-nat FP3	CGCTCTACATGAGCATGCCCTGCCCCTGATATGGTCTCTCTGATGCTGG
#5567	hex-1 KO RP4	CGGTTCAGGATGAGATGACC
**KI constructs**		
rrp-3-3xFLAG::hph		
#5748	rrp-3 KI FP1	ACGACGATGATGGAGACGAC
#5749	rrp-3 KI RP2	CCTCCGCCTCCGCCTCCGCCGCCTCCGCCATGCCTCTGGTAAATCTGCA
#5750	rrp-3 KI FP3	TGCTATACGAAGTTATGGATCCGAGCTCGCGGGGATTCAGAGGTTGAAG
#5751	rrp-3 KI RP4	GTAGGAAGCTAACGAGGGTG
dcl-2xFLAG::hph		
#3128	DCL-2 cPCR FP2	TCGTCGTCAAGCGTCTCATG
#5755	dcl-2 KI RP2	CCTCCGCCTCCGCCTCCGCCGCCTCCGCCGTTCATGACTGTATCCCCAT
#5756	dcl-2 KI FP3	TGCTATACGAAGTTATGGATCCGAGCTCGCAAGGGACAGTCTCTACTCC
#5366	DCL-2 KO RP4	AGTCCTTGTAAGGATGTGTGG
qde-2-3xFLAG::hph		
#5757	qde-2 KI FP1	AGGAACTCATGAGCTCGCAC
#5758	qde-2 KI RP2	CCTCCGCCTCCGCCTCCGCCGCCTCCGCCGATATAGTACATGGAGTTCC
#5759	qde-2 KI FP3	TGCTATACGAAGTTATGGATCCGAGCTCGGTGCTGGAATGTACTGGAGC
#5560	qde-2 KO RP4	ACAACTGGAACAGCTTTGTCG
**Northern probe preparation**		
rrp-3		
	NC-rrp3-2278_F	ACTGGAAGCCAAGAACAGG
	NC-rrp3-2798_R	TGGGATAGCCCTTGGATT
dcl-2		
	NC-dcl2-1191_F	CCTTGCTTCCCATCAACA
	NC-dcl2-1898_R	GCTGGCAGATGAACAGTTG

Supplementary Table 4. GenBank accession numbers of viral sequences presented in the phylogenetic trees

Genus	Virus Name	Abbreviation	Accession Number
fusariviruses	Fusarium poae fusarivirus 1	FpFV1	NC_030868
	Pleospora typhicola fusarivirus 1	PtFV1	NC_028470
	Rosellinia necatrix fusarivirus 1	RnFV1	NC_024485
	Penicillium roqueforti ssRNA mycovirus 1	PrMV1	NC_024699
	Fusarium graminearum dsRNA mycovirus-1	FgV1	NC_006937
	Penicillium aurantiogriseum fusarivirus 1	PaFV1	NC_028467
	Sclerotinia sclerotiorum fusarivirus 1	SsFV1	NC_027208
	Alternaria brassicicola fusarivirus 1	AbFV1	NC_029056
	Valsa ceratosperma hypovirus 1	VcHV1	NC_017099
	Sclerotinia sclerotiorum hypovirus 1	SsHV1	NC_015939
	Macrophomina phaseolina single-stranded RNA virus 1	MpRV1	KP900890
	Nigrospora oryzae fusarivirus 1	NoFV1	KU980909
hypoviruses	Cryphonectria hypovirus 4	CHV4	NC_00643
	Cryphonectria hypovirus 3	CHV3	NC_000960
	Cryphonectria hypovirus 2	CHV2	NC_003534
	Cryphonectria hypovirus 1	CHV1	NC_001492
partitiviruses (RdRp)	Aspergillus ochraceous virus	AoV	EU118277
	Atkinsonella hypoxylon virus	AhV	L39125
	beet cryptic virus 1	BcV1	EU489061
	beet cryptic virus 2	BcV2	HM560703
	cannabis cryptic virus	Cannabis_cV	JN196536
	carrot cryptic virus	Carrot_cV	FJ550604
	Ceratocystis resinifera partitivirus	CrPV	AY603052
	Amasya cherry disease associated partitivirus	AcPV	AJ781168
	Chondrostereum purpureum cryptic virus 1	CppV1	AM999771
	crimson clover cryptic virus 2	CccV2	JX971982
	dill cryptic virus 2	DcV2	JX971984
	Discula destructiva virus 1	DdV1	AF316992
	Discula destructiva virus 2	DdV2	AY033436
	fig cryptic virus	FcV	FR687854
	Flammulina velutipes browning virus	FvbV	AB465308
	Fusarium poae virus 1	FpV1	AF047013
	Fusarium solani virus 1	FsV1	D55668
	Gremmeniella abietina RNA virus MS1	GaV_MS1	AY089993
	Heterobasidion partitivirus 12	HetPV12	KF963175
	Heterobasidion partitivirus 13	HetPV13	KF963177
	Heterobasidion partitivirus 15	HetPV15	KF963186
	Heterobasidion RNA virus 1	HetRV1	HQ541323
	Heterobasidion partitivirus 2	HetRV2	HM565953
	Heterobasidion RNA Virus 3	HetRV3	FJ816271
	Heterobasidion partitivirus 7	HetPV7	JN606091
	Heterobasidion partitivirus 8	HetPV8	JX625227
	hop trefoil cryptic virus 2	HtcV2	JX971980
	Ophiostoma himal-ulmi partitivirus 1	OhPV1	AM087202
	Penicillium stoloniferum virus F	PsV-F	AY738336
	Penicillium stoloniferum virus S	PsV-S	AY156521
	pepper cryptic virus 1	PcV1	JN117276
	pepper cryptic virus 2	PcV2	JN117278
	Pleurotus ostreatus virus 1	PoV1	AY533038
	Primula malacoides virus 1	PmV1	EU195326
	red clover cryptic virus 2	RccV2	JX971978
	Rhizoctonia solani 717 partitivirus	RsPV	AF133290
	Rosellinia necatrix partitivirus 2	RnPV2	AB569997
	Rosellinia necatrix partitivirus 1	RnPV1	AB113347
	vicia cryptic virus	VcV	AY751737
	white clover cryptic virus 1	WccV1	AY705784
	white clover cryptic virus 2	WccV2	JX971976
	Cryptosporidium parvum virus 1	CpV1	U95995
	Aspergillus ochraceous virus	AoV_CP	EU118278
	Atkinsonella hypoxylon virus	AhV_CP	L39126
	beet cryptic virus 1	BcV1_CP	EU489062
	beet cryptic virus 2	BcV2_CP	HM560704
	cannabis cryptic virus	Cannabis_cV_CP	JN196537
	carrot cryptic virus	Carrot_cV_CP	FJ550605
	Ceratocystis resinifera partitivirus	CrPV_CP	AY603051
	Amasya cherry disease associated partitivirus	AcPV_CP	AJ781167
	Chondrostereum purpureum cryptic virus 1	CppV1_CP	AM999772
	crimson clover cryptic virus 2	CccV2_CP	JX971983
	dill cryptic virus 2	DcV2_CP	JX971985
	Discula destructiva virus 1	DdV1_CP	AF316993

partitiviruses (CP)	Discula destructiva virus 2	DdV2_CP	AF316994
	fig cryptic virus	FcV_CP	FR687855
	Flammulina velutipes browning virus	FvbV_CP	AB465309
	Fusarium poae virus 1	FpV1_CP	AF015924
	Fusarium solani virus 1	FsV1_CP	D55669
	Gremmeniella abietina RNA virus MS1	GaV_MS1_CP	AY089994
	Heterobasidion partitivirus 12	HetPV12_CP	KF963176
	Heterobasidion partitivirus 13	HetPV13_CP	KF963178
	Heterobasidion partitivirus 15	HetPV15_CP	KF963187
	Heterobasidion RNA virus 1	HetRV1_CP	HQ541324
	Heterobasidion partitivirus 2	HetRV2_CP	HM565954
	Heterobasidion RNA Virus 3	HetRV3_CP	FJ816272
	Heterobasidion partitivirus 7	HetPV7_CP	JN606090
	Heterobasidion partitivirus 8	HetPV8_CP	JX625228
	hop trefoil cryptic virus 2	HtcV2_CP	JX971981
	Ophiostoma himal-ulmi partitivirus 1	OhPV1_CP	AM087203
	Penicillium stoloniferum virus F	PsV-F_CP	AY738337
	Penicillium stoloniferum virus S	PsV-S_CP	AY156522
	pepper cryptic virus 1	PcV1_CP	JN117277
	pepper cryptic virus 2	PcV2_CP	JN117279
	Pleurotus ostreatus virus 1	PoV1_CP	AY533036
	Primula malacoides virus 1	PmV1_CP	EU195327
	red clover cryptic virus 2	RccV2_CP	JX971979
	Rhizoctonia solani 717 partitivirus	RsPV_CP	AF133291
	Rosellinia necatrix partitivirus 2	RnPV2_CP	AB569998
	Rosellinia necatrix partitivirus 1	RnPV1_CP	AB113348
	vicia cryptic virus	VcV_CP	AY751738
	white clover cryptic virus 1	WccV1_CP	AY705785
	white clover cryptic virus 2	WccV2_CP	JX971977
	Cryptosporidium parvum virus 1	CpV1_CP	U95996
